# Seawater Splitting Using NiFeP-Embedded Porous Carbon Fibers

**DOI:** 10.1021/acsaenm.5c00785

**Published:** 2025-11-07

**Authors:** Akshara Paras Parekh, Ashish Kumar Yadav, Brendan Whitfield, Yue Zhang, Arshad Aijaz, Guoliang Liu

**Affiliations:** † Department of Chemistry, 1757 Virginia Tech, Blacksburg, Virginia 24061, United States; ‡ Department of Energy & Human Sciences, 199276 Rajiv Gandhi Institute of Petroleum Technology, Jais, Amethi, Uttar Pradesh 229304, India; § Macromolecules Innovation Institute, Virginia Tech, Blacksburg, Virginia 24061, United States; ∥ Division of Nanoscience, Academy of Integrated Science, Virginia Tech, Blacksburg, Virginia 24061, United States

**Keywords:** electrocatalysts, transition metal phosphides, electrospinning, oxygen evolution reaction, hydrogen evolution reaction, water/seawater splitting

## Abstract

Designing efficient and durable nonprecious metal-based electrocatalysts for high-performance seawater splitting is essential for clean energy conversion. Nevertheless, the underlying cause of NiFeP activity, particularly in the oxygen evolution reaction (OER), remains poorly understood. Using a flexible and controllable electrospinning approach, we effectively synthesized bimetallic NiFeP encapsulated in porous carbon nanofibers (PCF). Benefiting from the strong synergistic effects of the mesoporous structures with optimized binary metal components encapsulated in the carbon nanofibers, NiFeP-PCFs exhibit improved OER and hydrogen evolution reaction (HER) performance in alkaline, neutral, and alkaline seawater electrolytes. The electrode exhibits overpotentials of 320 mV and 145 mV at 100 mA cm^–2^ for OER and HER, respectively, in 1 M KOH, with excellent durability (100 h at 100 mA cm^–2^). Furthermore, NiFeP-PCF requires a voltage of 1.8 V at 100 mA cm^–2^ for seawater splitting and can operate stably for over 200 h. Our research holds significant potential for developing efficient non-noble-metal bimetallic electrodes for water and seawater electrolysis.

## Graphical abstract

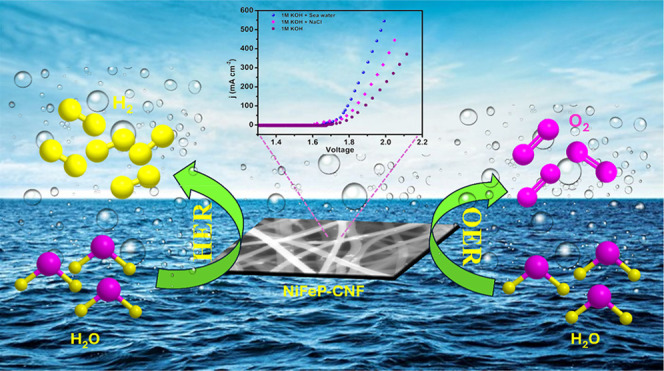

## 1 Introduction

The quest for affordable, sustainable, and reliable energy is one of the most critical challenges in modern society.^1–3^ As a promising energy storage medium, hydrogen is highly attractive due to its clean nature and the ability to be obtained through electrochemical water splitting.^4,5^ Hydrogen is considered sustainable when it is obtained using renewable resources, such as solar and wind power, which are intermittent yet environmentally friendly.^6^ Typically, electrochemical water splitting involves two half-reactions: the oxygen evolution reaction (OER) and the hydrogen evolution reaction (HER). The efficiencies of these reactions are constrained by their kinetics and energy barriers.^7,8^ Noble metal catalysts (e.g., Ru, Ir, and Pt) are commonly used in OER and HER reactions, but they are expensive, scarce in nature, and have low durability, which limits their large-scale commercialization.^9,10^ Therefore, there is an urgent need to develop inexpensive, accessible, durable, and efficient OER and HER catalysts for water electrolysis.

Compared to freshwater electrolysis, direct saltwater electrolysis has attracted substantial interest thanks to the abundance of seawater and its compatibility with expansive offshore renewable energy systems.^11,12^ However, the high salinity and composition complexity of seawater present significant hurdles. OER and chlorine oxidation reaction(s) (ClOR) have similar thermodynamic potentials, and the two reactions compete at the anode, reducing the water electrolysis efficiency. Thus, new OER electrocatalysts must have low overpotentials for OER. In practical seawater electrolysis, the main challenge is the competing chlorine evolution reaction (ClOR), which occurs at thermodynamic potentials close to OER and can compromise efficiency and catalyst stability. In alkaline environments, hypochlorite formation takes place at a potential ∼480 mV higher than OER,^13^ creating a window where OER selectivity can be preserved.^14,15^ To be effective, electrocatalysts must operate at low overpotentials while resisting chloride-induced corrosion and other impurities present in seawater. Thus, designing catalysts that combine high activity with long-term durability in chloride-rich conditions is critical for enabling practical seawater splitting.

Alternative to noble metals, transition metal phosphides (TMPs) are attractive low-cost candidates with tunable electronic properties.^16–18^ They usually have high electrical conductivity due to the electon-withdrawing nature of the electronegative P atoms.^19,20^ Particularly, transition metal alloy phosphides, composed of two or more distinct TMPs, can have enhanced OER and HER performances^21–23^ because of the synergistic effects among the constituent TMPs.^24,25^ Notably, nickel–iron phosphide (NiFeP) has been recognized for its OER and HER activities, rekindling interest as a candidate to replace noble metal catalysts.^26,27^ However, bimetallic phosphide particles are prone to corrosion and acidic deterioration during the catalytic processes.^28^ Encapsulating TMPs in a porous carbon scaffold is an effective approach to address the issue. The porous carbon scaffold can shield the TMP particles, preventing aggregation, corrosion, and deactivation, which results in improved catalytic properties.^29,30^ Among the various types of carbon scaffolds, one-dimensional carbon fibers are enticing electrocatalyst supports thanks to their high-temperature tolerance, electrical conductivity, mechanical strength, and flexibility.^31–33^ To prepare porous carbon fibers, researchers have utilized acids (e.g., HNO_3_) and bases (e.g., KOH) to chemically exfoliate conventional carbon fibers.^34^ However, these methods lack consistency and control over pore size, which negatively affects fiber integrity and electrochemical performance. Alternatively, researchers have prepared porous carbon fibers by coupling polyacrylonitrile (PAN) and pitch-based precursors with hard templates,^35^ soft templates,^36^ and sacrificial polymers^37^ to generate meso- and macro-pores.^38,39^ Although block copolymer synthesis and electrospinning are more complex than conventional methods, recent advances in high-throughput and continuous electrospinning make scalable production feasible. The self-assembly of block copolymers enables uniform confinement of active nanoparticles, enhancing catalytic performance compared to traditional approaches. This demonstrates that block copolymer-derived porous fibers are a practical and effective route for high-performance electrocatalysts.

While NiFeP-based electrocatalysts have been extensively reported, the primary novelty of this work lies in employing block copolymer self-assembly to fabricate porous carbon fibers with uniform mesopores and narrow pore distributions.^40,41^ This templating approach enables precise confinement and stabilization of NiFeP nanoparticles within the conductive carbon framework, thereby increasing the accessible surface area, accelerating electron and mass transport, and improving structural integrity during long-term operation. Unlike conventional carbonization or etching methods, this design distinguishes our approach as a key merit of the study. Block copolymers, as opposed to homopolymer blends, microphase separate^42^ into homogeneous nanostructures of 2–50 nm. Upon pyrolysis, electrospun PAN-based block copolymer fibers generated structures with interconnected hierarchical micro-, meso-, and macro-pores.^43,44^ The characteristics of one-dimensional fibers (e.g., doping and surface layer thickness) can be adjusted by tuning the electrospinning conditions.^45,46^ Herein, we employ PAN-based block copolymers to synthesize one-dimensional porous carbon fibers (PCF) loaded with NiFeP. The resulting NiFeP-PCF exhibited good performance under neutral and alkaline conditions for OER and HER. Arranging NiFeP-PCFs as both anode and cathode catalysts in water splitting afforded a low cell voltage of 1.79 V at 100 mA cm^–2^ and provided a nearly uniform Faradaic efficiency.

## 2 Experimental Section

### 2.1 Materials

Analytical-grade chemicals were all used as received without further processing. Acrylonitrile (AN; ≥99%) and methyl methacrylate (MMA; ≥99%) were passed through an alumina column prior to use to remove inhibitors. Cumyl dithiobenzoate (CDB; ≥99%) was synthesized according to the literature.^1^ 2,2′-azobis­(2-methylpropionitrile) (AIBN; ≥98%), benzene (≥99.9%), aluminum oxide (activated, neutral, Brockmann Activity I), *N*,*N*-dimethylformamide (DMF; ≥99.7%), Ni­(II) acetylacetonate (Ni-(acac)_2_) and Fe­(III) acetylacetonate (Fe­(acac)_3_), phosphorus pentoxide (P_2_O_5_) were purchased from Sigma-Aldrich and used as received. Pt/C (20 wt %) and IrO_2_ commercial catalysts were obtained from Johnson Matthey and Alfa, respectively, and used as received.

### 2.2 Synthesis of PAN-*b*-PMMA Block Copolymer

Reversible addition–fragmentation chain-transfer (RAFT) polymerization was used to synthesize macro-poly­(methyl methacrylate) (PMMA) and poly­(acrylonitrile-*b*-methyl methacrylate) (PAN-*b*-PMMA) block copolymer. First, a 100 mL Schlenk flask was charged with MMA (35.0 mL, 310 mmol), CDB (84.3 mg, 0.309 mmol), and AIBN (25.4 mg, 0.155 mmol) dissolved in benzene (51.6 mL). The solution was subjected to three freeze–pump–thaw (FPT) cycles and backfilled with N_2_. The flask was set in an oil bath at 60 °C and stirred for 24 h. The resultant PMMA macro-chain transfer agent was precipitated in methanol and dried in vacuo at 60 °C for 12 h. To synthesize PAN-*b*-PMMA, macro-PMMA (0.65 g, 11 mmol), acrylonitrile (2.6 mL, 43 mmol), AIBN (0.44 mg, 2.7 mmol), and DMSO (7.22 mL) were mixed in a 40 mL Schlenk flask with a magnetic stir bar. The solution was subjected to three FPT cycles and then backfilled with N_2_. The solution was placed in an oil bath at 65 °C and stirred for 24 h. The resulting PAN-*b*-PMMA was precipitated in methanol and dried in vacuo for 12 h.

### 2.3 Preparation of NiFeP-Doped Porous Carbon Fibers

Initially, 2.25 mmol of Ni­(acac)_2_, 1.25 mmol of Fe­(acac)_3_, phosphorus pentoxide, and PAN-*b*-PMMA were dissolved in 10 mL of DMF with constant overnight stirring at 70 °C. The solution was transferred to a disposable syringe and electrospun at a voltage of 18 kV and a feeding rate of 1.5 mL/h, and collected on a rotating aluminum drum. Following electrospinning, the fiber mat was removed and dried in vacuo at 60 °C for 6 h. The fibers were oxidized at 280 °C for 8 h (ramp rate, 1 °C/min) in a tube furnace under ambient conditions. The oxidized fiber mat was then pyrolyzed at 800 °C (ramp rate, 10 °C/min) for 1 h in an N_2_ flow. NiP-, FeP-, and NiFe-PCF were synthesized similarly, utilizing the respective inorganic precursors.

## 3 Results and Discussion

Initially, PAN-*b*-PMMA (112-*b*-46 kDa) was synthesized via RAFT polymerization and used as the carbon precursor. The molecular weights of PMMA (number-average molecular weight, *M*_n_ = 46 kDa; dispersity, *D̵* = 1.08) and PAN-*b*-PMMA (*M*_n_ = 158 kDa, *D̵* = 1.13) were measured by ^1^H NMR and size exclusion chromatography (SEC) (Figure S1). PAN-*b*-PMMA nanofibers acted as substrates for loading active catalyst precursors, i.e., Ni­(acac)_2_, Fe­(acac)_3_, and phosphorus pentoxide, during electrospinning. The as-spun nanofibers were oxidized at 250 °C in air and pyrolyzed at 800 °C in N_2_ to generate PCF with active NiFeP catalysts (Figure 1).

**Figure 1:**
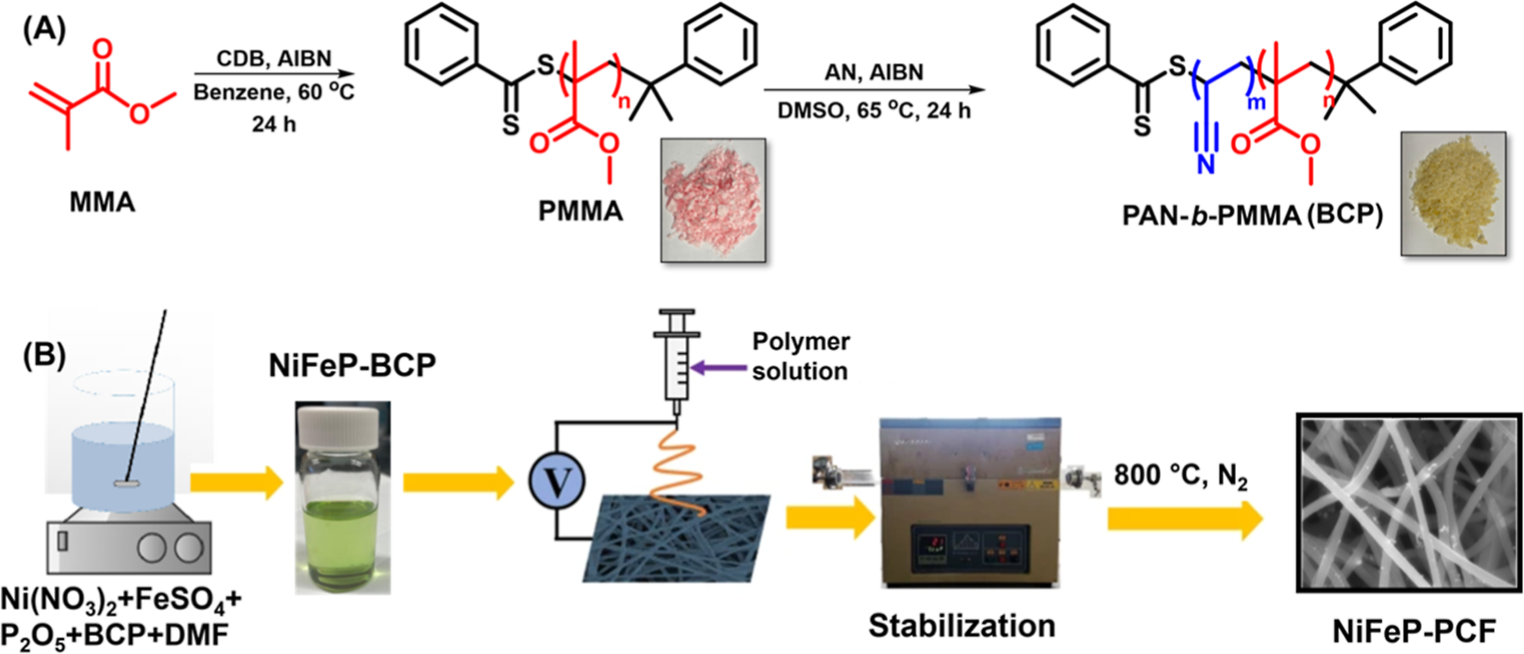
Synthesis of (A) PAN-*b*-PMMA via RAFT polymerization and (B) NiFeP-PCF via electrospinning and pyrolysis.

The NiFeP-PCF nanofibers exhibited a uniform diameter and smooth surfaces, forming an interconnected three-dimensional network (Figure 2a,b). Scanning electron microscopy (SEM) revealed that nanoparticles were uniformly distributed within the fibers, with a few additional particles present on the exterior surface of the PCFs. Similarly, NiFe-, NiP-, and FeP-PCF displayed a smooth surface and uniform fiber diameters (Figure S2). High-resolution transmission electron microscopy (HRTEM) images of NiFeP-PCF showed that the NiFeP nanoparticles were embedded in PCF (Figure 2c–f). The porous structure, both internally and on the surface, provided a large specific surface area, which is advantageous for exposing a greater number of active sites for electrochemical catalysis. As illustrated in Figure 2c, NiFeP nanoparticles were anchored onto the carbon nanofiber surface and were encapsulated by graphitic carbon shells. These shells were formed from the carbon matrix, catalyzed by NiFeP during high-temperature treatment. The nanoparticles exhibit an average size in the range of 17–22 nm. The particle size distribution, shown in the inset of Figure 2d, follows a log-normal function, which is typical for nanoparticle systems. The HRTEM image (Figure 2f) displayed distinct lattice fringes with a spacing of 0.21 nm, attributed to the (111) plane of the face-centered cubic (fcc) NiFeP phase. The encapsulation of NiFeP nanoparticles within graphitic carbon shells not only shields the alloy from corrosion under harsh conditions but also enhances the electrocatalytic performance of the carbon matrix due to partial electron transfer from the metal core to the surrounding carbon.^47^ Additionally, EDS elemental mapping (Figure 2g) confirmed the uniform distribution of C, N, O, Ni, Fe, and P elements throughout the nanofiber composite. This unique porous structure is potentially beneficial for exposing more active sites, leading to improved electrocatalytic performance.

**Figure 2:**
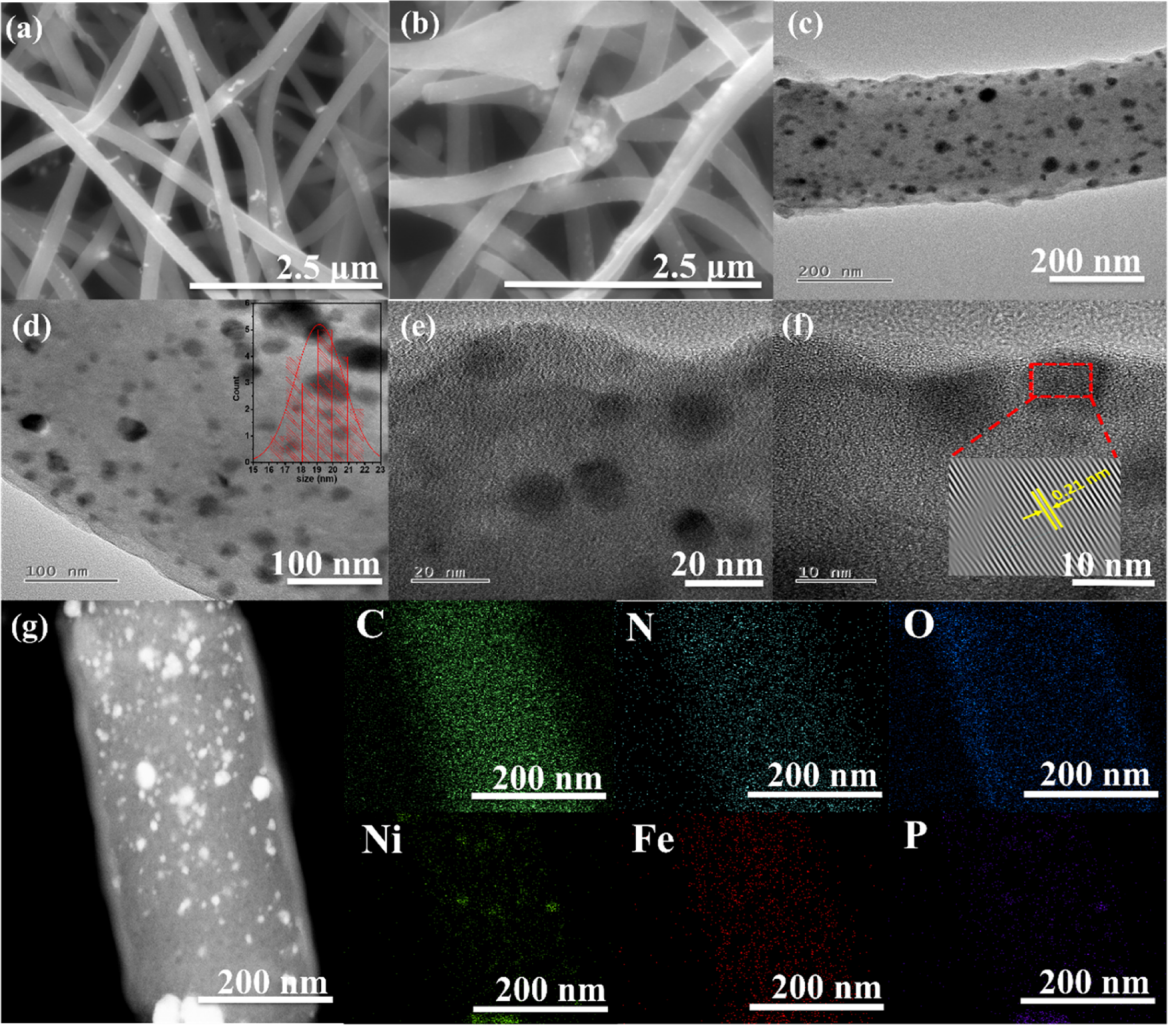
(a,b) SEM images, (c–f) HR-TEM images, (d) inset image of particle size distribution histogram, (f) inset image showing fringe width of NiFeP nanoparticles, and (g) HAADF-STEM image of NiFeP-PCF with the corresponding elemental mappings of C, N, O, Ni, Fe, and P.

Powder X-ray diffraction (XRD) and X-ray photoelectron spectroscopy (XPS) were employed to characterize the phase structures and chemical compositions of the catalysts (Figure 3). XRD showed that NiFeP-, NiFe-, NiP-, and FeP-PCF all possessed a characteristic peak at 25.7° after pyrolysis corresponding to the (002) plane of carbon (Figure 3a).^48^ The XRD pattern of NiFeP-PCF showed a combination of Ni_2_P (PDF no. 27-1171)^49^ and Fe_2_P (PDF no. 71-2262),^50^ indicating the successful synthesis of a TMP alloy catalyst of Ni_2_P and Fe_2_P on PCF. The surface areas and pore size distributions were determined using N_2_ sorption isotherms based on the Brunauer–Emmett–Teller (BET) theory and nonlocal density functional theory (NLDFT), respectively. N_2_ adsorption/desorption isotherms of pure PCF and NiFe-, FeP-, NiP-, and NiFeP-PCF showed typical type IV characteristics (Figure 3b), displaying a prominent hysteresis loop suggestive of mesoporous structures.^51^ The measured surface area (*S*_BET_) of the pure PCF was 576.52 m^2^ g^–1^, higher than that of FeP-PCF (418.64 m^2^ g^–1^), NiP-PCF (352.97 m^2^ g^–1^), NiFe-PCF (309.53 m^2^ g^–1^), and NiFeP-PCF (251.42 m^2^ g^–1^). *S*_BET_ decreased likely due to the introduction of nanoparticles to the porous carbon matrix. According to the NLDFT pore-size distribution, the mesopores were in the range of 2–50 nm (Figure S3). The relatively large specific surface area and high pore volume would allow gaseous products to diffuse quickly, aiding in fast mass transfer and intimate contact between the electrolytes and catalysts, and thus providing improved electrocatalytic activity. Raman spectra of the synthesized catalysts were recorded to probe their structural features and degree of graphitization (Figure S4). Two distinct peaks can be observed in all the curves, which are attributed to the crystalline graphic carbon structure (G band) and disordered amorphous carbon structure (D band). The intensity ratio (*I*_D_/*I*_G_) reflects the defect density and graphitization level of the carbon matrix. The PCF showed the lowest *I*_D_/*I*_G_ ratio, indicating a higher degree of graphitization, while the incorporation of metal and metal phosphides (NiP-PCF, FeP-PCF, NiFe-PCF, NiFeP-PCF) progressively increased the I_D_/I_G_ ratio, suggesting enhanced structural defects due to nanoparticle integration. These generated defects could promote the active site exposure and improve the electrocatalytic efficiency.^52,53^

**Figure 3:**
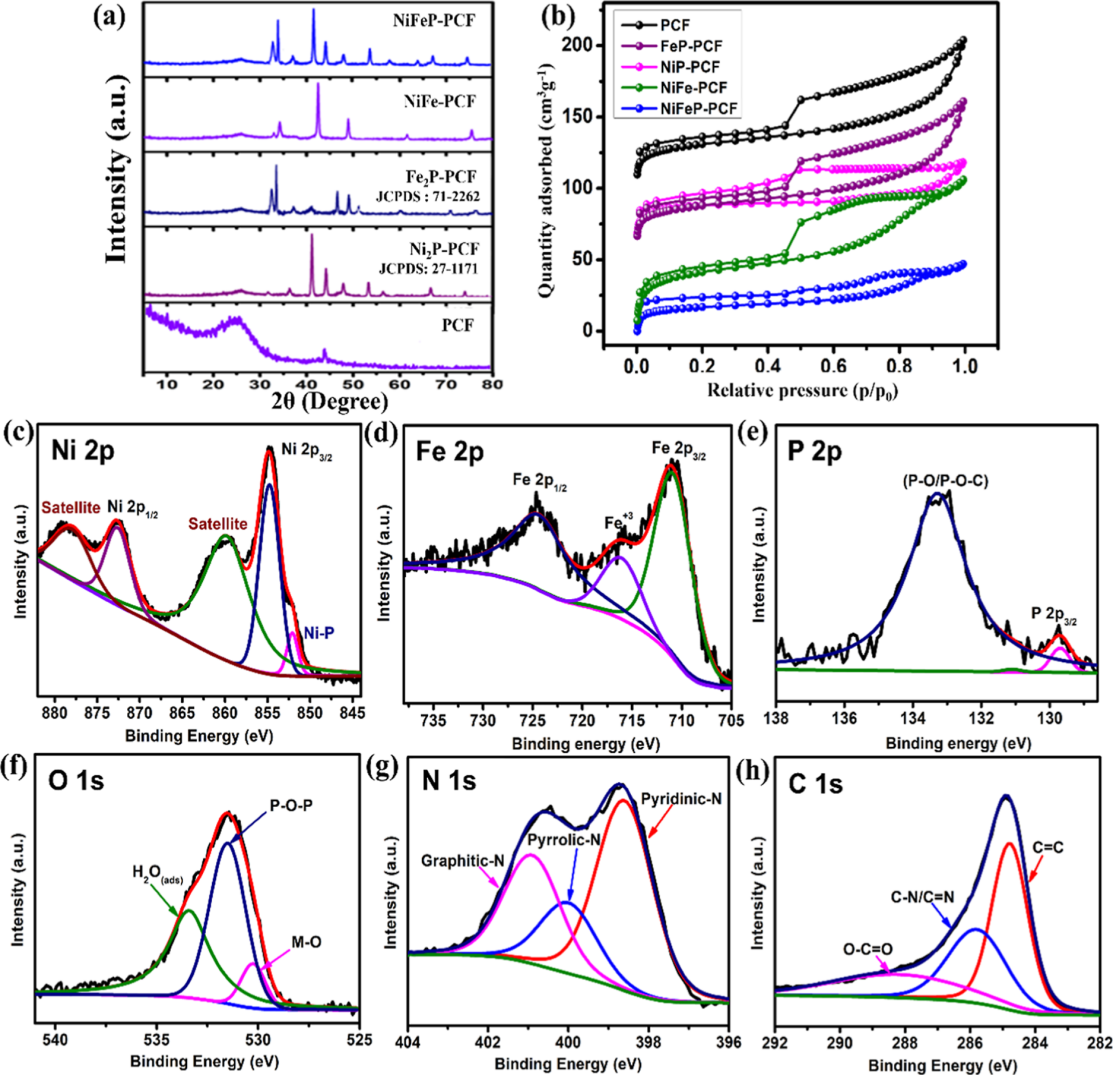
(a) XRD patterns, and (b) N_2_ adsorption/desorption isotherms of PCF, NiP-PCF, FeP-PCF, NiFe-PCF, and NiFeP-PCF. (c–h) XPS spectra of (c) Ni 2p, (d) Fe 2p, (e) P 2p, (f) O 1s, (g) N 1s, and (h) C 1s of NiFeP-PCF.

The surface composition of NiFeP-PCF, as determined by XPS, confirmed the coexistence of Ni, Fe, P, C, N, and O. High-resolution XPS spectra of Ni 2p of NiFeP-PCF (Figure 3c) displayed five peaks. In the Ni 2p_3/2_ region, a primary peak at 854.9 eV and a satellite peak at 860.0 eV were observed, while in the Ni 2p_1/2_ region, a primary peak at 872.6 eV and a satellite peak at 878.4 eV were detected, which are consistent with Ni^2+^ species. In addition, the existence of a weak peak at 852.1 eV suggests that (NiFe)_2_P contains Ni–P bonds. The Fe 2p spectra of NiFeP-PCF (Figure 3d) showed Fe 2p_3/2_ and Fe 2p_1/2_ peaks at 711.1 and 724.6 eV, which are related to the oxidized peak of Fe with a satellite peak at 716.4 eV.^54^ In the high-resolution P 2p spectra (Figure 3e), the peak at 129.7 eV is assigned to the phosphorus in metal phosphide. The peak at 133.3 eV corresponded to the oxidized phosphorus in phosphate.^55^ In the O 1s spectra (Figure 3f), a peak at 531.5 eV was attributed to P–O (nonbridging oxygen), further corroborating the existence of oxidized surface metal phosphates. In addition, the peaks at 530.2 and 533.4 eV corresponded to C–O and surface-adsorbed water molecules, respectively.^56^ The high-resolution N 1s spectrum (Figure 3g) revealed three distinct peaks at 398.6, 400.1, and 400.9 eV, corresponding to pyridinic, pyrrolic, and graphitic nitrogen, respectively. According to a previous report,^57^ the pyridinic-N and pyrrolic-N species are essential for enhanced HER and OER activities because they serve as metal coordination sites and active sites, while graphitic-N retains the electrical conductivity of carbon hybrids. As mentioned earlier, carbon elements are expected to be found in graphene-like carbon structures, which is consistent with the C 1s spectrum analysis (Figure 3h). The above analysis demonstrates that NiFeP-PCF was successfully synthesized, and a P bridge linking the carbon layer and TMPs may exist. Furthermore, XPS analysis confirms the general presence of Ni, Fe, and P species on the catalyst surface, indicating that partial surface oxidation of phosphides occurs under ambient conditions. These surface states can dynamically reconstruct into Ni/Fe (oxy)­hydroxides during electrocatalysis, serving as the real active phase. This observation is consistent with recent reports, highlighting the crucial role of surface electronic modulation and the synergistic interaction among Ni, Fe, and P in enhancing electrocatalytic activity.^58,59^

### 3.1 Electrocatalytic Performance in Alkaline Media

#### 3.1.1 OER

In electrocatalysis, the proximity of active sites to a porous carbon matrix facilitates mass and electron transfer. The OER performance of the electrocatalysts herein was evaluated using a standard three-electrode configuration in 1.0 M KOH. Polarization curves of NiFeP-, NiFe-, NiP-, and FeP-PCF were compared with that of commercial IrO_2_ (Figure 4a). Among all catalysts, NiFeP-PCF showed excellent OER performance with a low overpotential of 340 mV at a current density of 100 mA cm^–2^ due to the porous structures revealed by microscopy and the metallic nature of the NiFeP that facilitates the electron transfer. This overpotential compares favorably to most non-noble-metal phosphides and oxides in the literature (Figure S5a). We also synthesized NiFe-PCF (400 mV), NiP-PCF (470 mV), and FeP-PCF (510 mV), showing that NiFeP-PCF was much more active than the other materials. This observation suggests that extrinsic bimetallic phosphide substitution may serve as a general strategy for enhancing electrocatalytic performance. The NiFeP-PCF even surpasses the commercial IrO_2_ (440 mV) for similar current density, which is considered a state-of-the-art catalyst.

**Figure 4:**
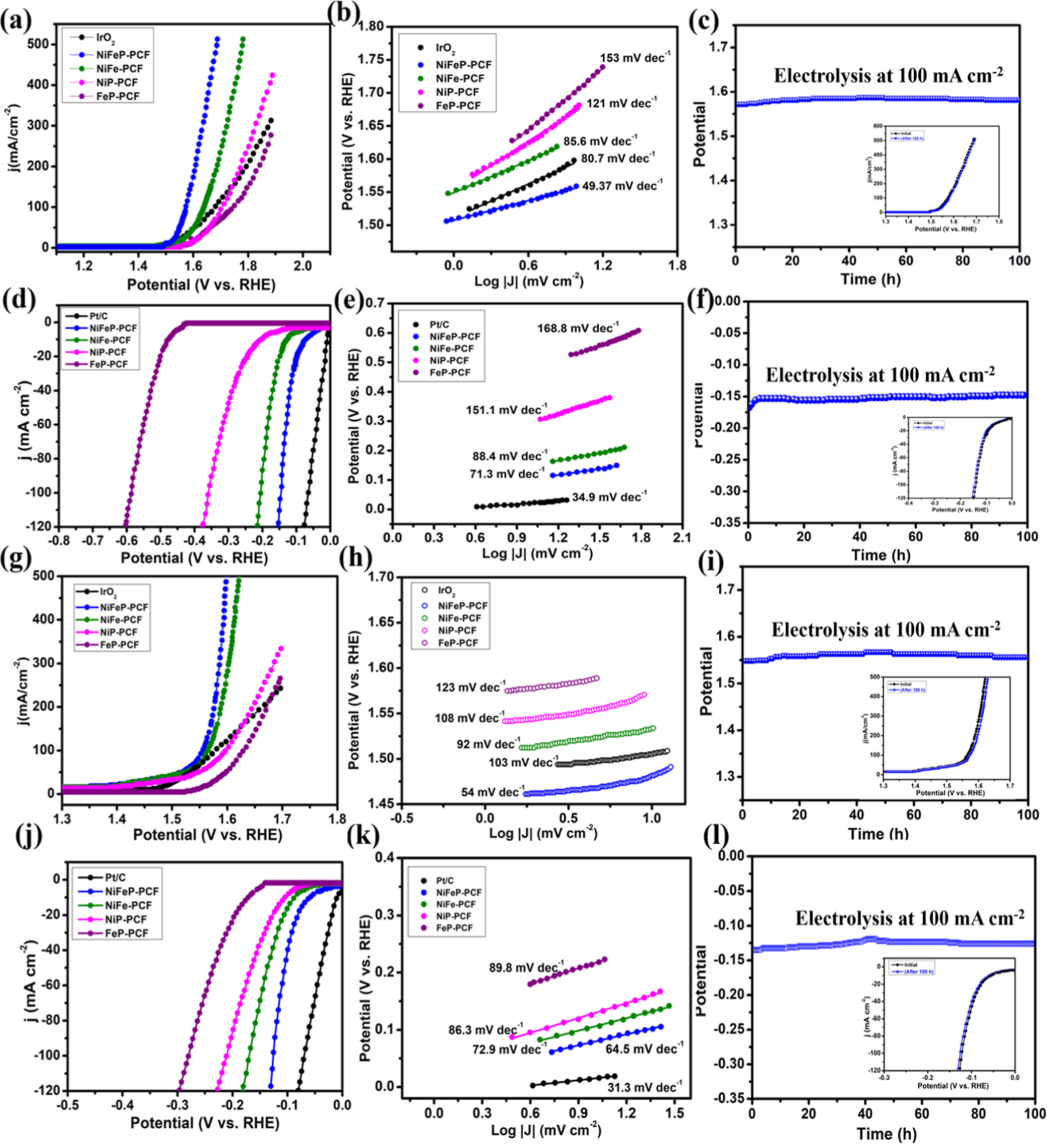
(a,d,g,j) LSV curves, (b,e,h,k) Tafel plots of as-synthesized electrocatalysts, and (c,f,i,l) chrono-potentiometric stability test of NiFeP/PCF @100 mA cm^–2^ for (a,b,c) OER in 1 M KOH, (d–f) HER in 1 M KOH, (g–i) OER in PBS (pH = 7), (j–l) HER in PBS with a scan rate of 5 mV s^–1^. IrO_2_ and Pt/C are included for comparison.

The OER reaction kinetics were assessed using the Tafel slope obtained from LSV measurements for various electrocatalysts. The NiFeP-PCF catalyst exhibited a notably small Tafel slope of 49.37 mV dec^–1^, compared to that of IrO_2_ (80.7 mV dec^–1^), known to be a state-of-the-art electrocatalyst for OER, along with NiFe-PCF (85.6 mV dec^–1^), NiP-PCF (121 mV dec^–1^), and FeP-PCF (153 mV dec^–1^). This low Tafel slope of NiFeP-PCF indicates superior electrocatalytic kinetics compared to the other catalysts investigated herein (Figure 4b). In addition, an exchange current density of 0.27 mA cm^–2^ suggests better catalytic and kinetic performance of NiFeP-PCF compared to other synthesized electrocatalysts. (Figure S5b). Charge transfer kinetics were further assessed using electrochemical impedance spectroscopy (EIS) at 1.5 V. The EIS spectrum of NiFeP-PCF showed the smallest semicircle and the lowest charge transfer resistance value of 2.2 Ω among all fabricated electrocatalysts. The lower charge resistance (*R*_ct_) value can be attributed to the enhanced conductivity and accelerated electron transfer kinetics (Figure S6). Another parameter to efficiently characterize and optimize the electrocatalytic activity is the electrochemical surface area (ECSA), which is proportional to the double-layer capacitance (*C*_dl_). Cyclic Voltammograms at different scan rates (20 −120 mV s^–1^) were acquired in the nonfaradaic zone to determine the *C*_dl_ values (Figure S7). The NiFeP-PCF demonstrated the highest *C*_dl_ value of 45.2 mF cm^–2^, outperforming all other catalysts examined in this study, relating its high activity toward OER to its efficient charge transfer mechanism (Figure S8a).

To evaluate the intrinsic catalytic activities, the turnover frequency (TOF) values were determined using previously reported methods^60–62^ for all electrocatalysts herein. Among all, the NiFeP-PCF showed the highest TOF value of 5.6 s^–1^, much greater than that of the rest catalysts (Figure S8b), indicating a high abundance of active sites for OER in the electrocatalyst structure. For an electrocatalyst to be implemented at a commercial scale, durability and long-term stability under extreme and harsh pH conditions are essential, in addition to high catalytic activity. Chronopotentiometry was employed to assess stability under a constant current density of 100 mA cm^–2^. NiFeP-PCF showed an almost negligible increase in the potential value (Figure 4c), retaining the catalytic activity and high durability for at least 100 h at a current density of 100 mA cm^–2^ under a harsh alkaline environment. The polarization curves before and after the chronopotentiometry analysis for the NiFeP-PCF exhibited minimal deviation (inset Figure 4c).

#### 3.1.2 HER

The HER activities were assessed under the alkaline conditions of 1.0 M KOH. NiFeP-PCF showed the most prominent HER activity with a minimal overpotential of 145 mV at a current density of 100 mA cm^–2^, much smaller than NiFe-PCF (205 mV), NiP-PCF (360 mV), and FeP-PCF (580 mV) but comparable to commercially available Pt/C (65 mV) (Figure 4d). The reaction dynamics of NiFeP-PCF were determined by its Tafel slope from LSV measurements. The considerably smaller Tafel slope of NiFeP-PCF (71.3 mV dec^–1^) indicated its faster HER kinetics than the rest of the electrocatalysts in this work: NiFe-PCF (88.4 mV dec^–1^), NiP-PCF (151.1 mV dec^–1^), and Fe–P (168.8 mV dec^–1^). From the Tafel slope values, NiFeP-PCF was predicted to follow the Volmer–Heyrovsky mechanism for HER, with electrochemical desorption of dihydrogen being the rate-determining step.^63^ As an indicator of the inherent HER activity, current exchange density (*j*_0_) was extracted from the Tafel plots (Figure S9a). The *j*_0_ value of NiFeP-PCF (0.71 mA cm^–2^) was much higher than that of NiFe-PCF (0.53 mA cm^–2^), NiP-PCF (0.46 mA cm^–2^), and FeP-PCF (0.18 mA cm^–2^). Additionally, NiFeP-PCF exhibited superior electrocatalytic activity, exhibiting a TOF of 3.7 s^–1^, much higher than that of the rest of the catalysts (Figure S9b). Meanwhile, NiFeP-PCF demonstrated superior charge transfer capability with a significantly low charge transfer resistance (3.3 Ω) compared to NiFe-PCF (7.7 Ω), NiP-PCF (8.6 Ω), and FeP-PCF (10.3 Ω) (Figure S10). A similar observation was made while determining its double-layer capacitance (C_dl_) to estimate ECSA of the catalysts (Figure S11). Remarkably, NiFeP-PCF displayed the highest *C*_dl_ (34.8 cm^2^) and the most accessible active sites for HER, compared to NiFe-PCF (31.4 cm^2^), NiP-PCF (27.3 cm^2^), and FeP-PCF (22.6 cm^2^) (Figure S12a), which was found to be in good agreement with the TOF value of NiFe-PCF (3.7 s^–1^) being higher than other developed electrocatalysts toward HER reactions. This overpotential compares favorably to most non-noble-metal phosphides and oxides in the literature (Figure S12b).

Additionally, NiFeP-PCF showed a remarkable stability in 1 M KOH toward HER, operating for over 100 h at a high current density of 100 mA cm^–2^, as shown by the chronoamperometry (CP) test (Figure 4f). To further analyze the effect of long-term operation, the polarization curves were compared before the chronoamperometric treatment and after the stability test. The polarization curves showed an almost negligible increase in overpotential value toward HER, suggesting strong adherence of the electrocatalysts to the surface of NiFe-PCF without leaching into the electrolyte.

### 3.2 Electrolysis of Water in Neutral Media

The high alkaline and low pH acidic conditions deteriorate electrocatalysts during water electrolysis. Most catalysts cannot withstand operations in these media at industrially relevant current densities. Neutral media water electrolysis becomes an alternative. NiFeP-PCF was assessed for its electrocatalytic activities (HER and OER) in a typical H-cell filled with phosphate-buffered solution (PBS) of pH 7. The polarization curve suggested highly efficient water electrolysis at low overpotentials of 125 mV and 320 mV for HER and OER, respectively, at a current density of 100 mA cm^–2^, as compared to commercially available IrO_2_ (350 mV) for OER and 20% Pt/C (68 mV) for HER.

NiFeP-PCF showed the fastest electrocatalytic reaction kinetics according to the Tafel slopes (OER, 54 mV dec^–1^; HER, 64.5 mV dec^–1^, Figure 4h,k). The Nyquist plots indicated that NiFeP-PCF had a low charge transfer resistance (OER, 3.2 Ω; HER, 2.9 Ω) compared to the other synthesized catalysts, indicating its superior charge transfer capabilities (Figure S13). Chronoamperometric studies revealed that NiFeP-PCF had an exceptional stability with minimal current density changes at 100 mA cm^–2^ over 100 h (Figure 4i,l) for both HER and OER reactions. To evaluate the efficiency of water splitting, a two-electrode system was constructed using a NiFeP-PCF-coated cathode and anode. The system displayed excellent catalytic activity in both OER and HER, as evidenced by a polarization curve in PBS electrolyte that reached a current density of 100 mA cm^–2^ for water splitting (Figure S14a). Furthermore, the NiFeP-PCF electrodes showed impressive electrochemical stability, sustaining a current density of 100 mA cm^–2^ over 100 h of continuous operation in PBS electrolyte (Figure S14b).

Apart from evaluating the electrocatalytic activity of NiFeP-PCF in alkaline and neutral pH solutions, we examined the electrocatalytic activity of NiFeP-PCF for OER and HER in simulated (1 M KOH +0.5 M NaCl) and seawater mixed KOH (viz, 1 M KOH + seawater) in a conventional three-electrode cell to test its potential operational for industrial-scale seawater electrolysis. The natural seawater was collected from Myrtle Beach, South Carolina, USA. To attain a current density of 100 mA cm^–2^, the catalyst required an overpotential of 250 mV, showing excellent catalytic activity toward OER in seawater as compared to in alkaline and simulated seawater electrolytes (Figure 5b). The HER performance was substantially improved for seawater electrolyte, achieving a current density of 100 mA cm^–2^ with a low overpotential of 121 mV only (Figure 5c). This increased electrocatalytic activity for OER and HER can be attributed to the presence of supporting electrolytes or an increased number of available ions in seawater and simulated seawater, which are present in the form of soluble ions or salts. This enhances mass transfer rates and, consequently, improves electrokinetic current density.

**Figure 5:**
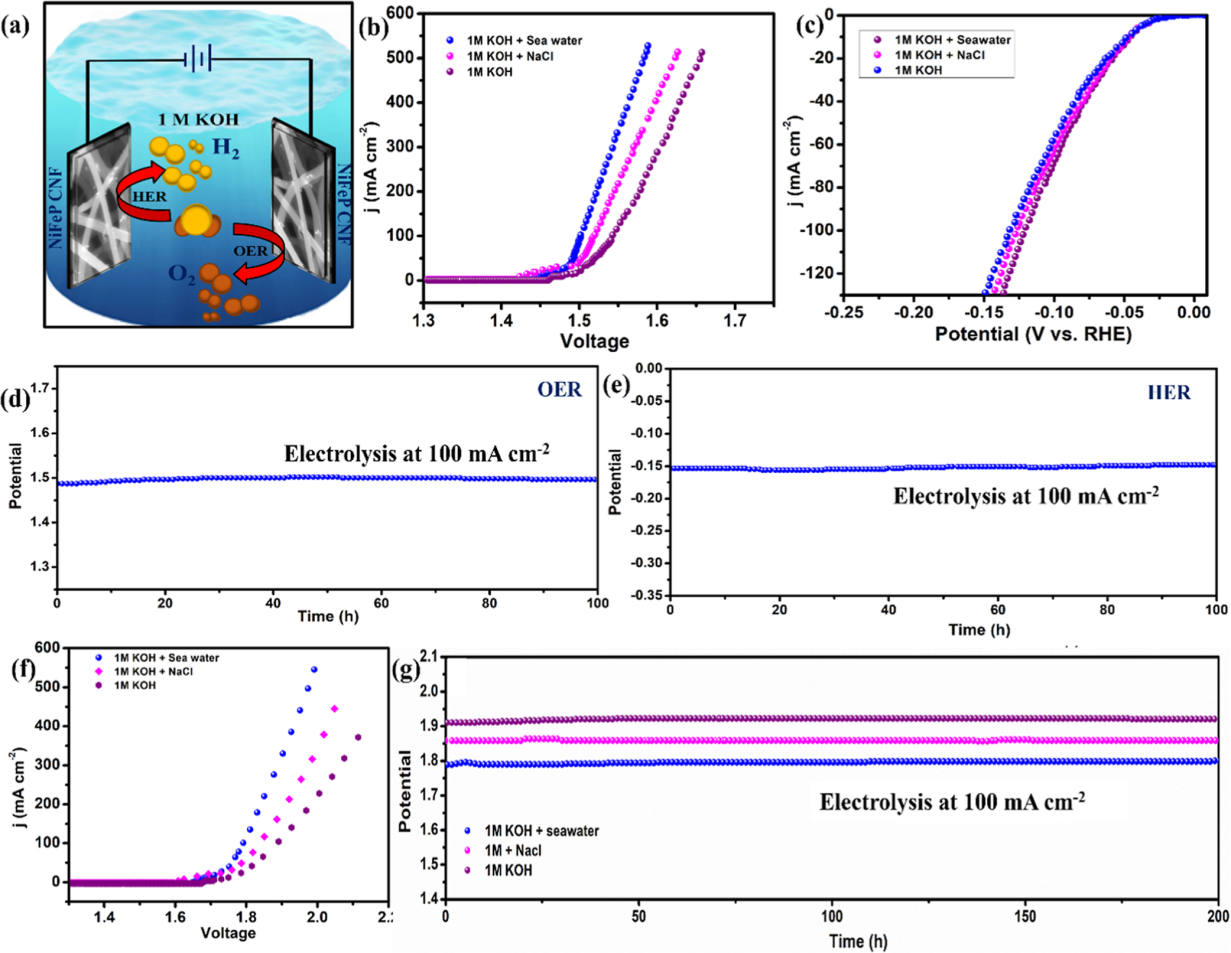
Seawater electrolysis in a three-electrode connection. (a) Schematic representation for seawater electrolysis, (b) OER LSV, (c) HER LSV in 1 M KOH, 1 M KOH mixed in 0.5 M NaCl, and 1 M KOH mixed with seawater, and (d,e) the corresponding chrono-potentiometric stability measurements in (1 M KOH + seawater) electrolyte of NiFeP-PCF electrode for OER and HER, respectively. Sea water electrolysis in a two-electrode setup: (f) LSV curve measured for seawater splitting compared in 1 M KOH, (1 M KOH+0.5 M NaCl), and (1 M KOH + seawater), and (g) their corresponding chrono-potentiometric cell voltage measurement for a current density of 100 mA cm^–2^ in various electrolytes up to 200 h.

The durability of NiFeP-PCF was assessed using chronopotentiometry (CP) in an alkaline seawater electrolyte in a three-electrode configuration, with separate tests conducted for 100 h each for OER and HER. After 100 h of operation at a current density of 100 mA cm^–2^ in 1 M KOH + seawater, we observed no increase in the required overpotentials, or signs of degradation such as leaching and active site poisoning, demonstrating excellent long-term stability for OER (Figure 5d). Likewise, NiFeP-PCF revealed high stability and displayed minimal potential decay for HER in alkaline natural seawater for >100 h at 100 mA cm^–2^ (Figure 5e). In addition, after 100 h of continuous seawater electrocatalysis measurements, the electrolyte was employed for the potassium iodide-starch paper test^64^ (Figure S15), and the impregnated seawater paper remained colorless, indicating that NiFeP-PCF exhibited both high activity and effective corrosion resistance. All these results demonstrated the remarkable OER and HER performance of NiFeP-PCF in (1 M KOH + seawater) electrolyte in a three-electrode setup in an H-cell.

### 3.3 Seawater Electrolysis in a Two-Electrode Setup

To evaluate the overall water-splitting efficiency, a two-electrode setup was established (in Figure 5a). The distance between the NiFeP-PCF cathode and anode was 1 cm. Inspired by the remarkable catalytic OER and HER performance, the polarization curve obtained in real seawater, i.e., 1 M KOH + seawater electrolyte, showed a current density of 100 mA cm^–2^ for water splitting at a very low cell voltage of 1.8 V (Figure 5f). NiFeP-PCF exhibited an exceptional electrochemical stability (Figure 5g), and the cell voltage remained consistent at a current density of 100 mA cm^–2^ for >200 h of nonstop operation in 1 M KOH mixed seawater electrolyte. We also investigated the potential formation of oxidized chloride species after long-term testing, and the absence of any color change in the reagent indicates that no side reactions occurred during seawater electrolysis. The electrolyzer Faradaic efficiency in alkaline seawater at ambient temperature was assessed and amounted to ∼97.8% by collecting the evolved gaseous products H_2_ and O_2_ (molar ratio = 2:1) over the cathode and anode, respectively, using the water displacement method (Figure S16). The fast electrokinetic response, long-term durability, and high faradaic efficiency highlight NiFeP-PCF as a good and viable candidate for the quest of electrocatalyst for alkaline seawater splitting at high current densities in harsh conditions.

To better understand the catalytically active sites of NiFeP-PCF responsible for its exceptional activity, we examined the nanostructure, surface composition, and chemical state after a stability test of seawater splitting for 200 h. SEM images of the catalyst after the tests (Figure S17a) showed that the NiFeP nanoparticles maintained their integrity in the carbon fibers, showcasing their exceptional structural robustness and superior resistance to corrosion. However, XRD showed the characteristic peaks corresponding to Ni_2_P and Fe_2_P have almost disappeared, and new peaks corresponding to NiOOH and FeOOH appeared, suggesting partial oxidation of Ni_2_P and Fe_2_P (Figure S17b) and surface reconstruction of phosphide into the oxy hydroxide. Additionally, XPS of NiFeP-PCF was also carried out after the stability test. In the Ni 2p spectra, the Ni–P peak (Figure S17c) diminished, and a new distinctive peak emerged at 874.6 eV, which is attributed to Ni­(III) due to the formation of NiOOH species.^65,66^ This change indicates that the Ni–P species can oxidize to form high-valence Ni–O species during the OER process. A broad peak centered at 714.8 eV in the Fe 2p spectra of NiFeP-PCF (Figure S17d) originated due to the formation of Fe­(III) in FeOOH,^67^ implying that NiFe oxyhydroxide might be the primary surface component. In the phosphorus 2p spectra, only the peak corresponding to the P–O bond was present (Figure S17e). Additionally, the peaks associated with M-P almost vanished, and the peak related to P–O (134.5 eV) shifted from 133.6 eV after the OER test.^67^ During the OER process, the positively charged metal atoms (Ni and Fe) can trap hydroxyl ions and generate active intermediates (NiFeOOH),^68^ as shown by XRD and XPS after the durability test. The surface P atoms were initially oxidized to hydroxides and subsequently converted to oxyhydroxides, which enhanced the adsorption and desorption processes during seawater splitting. To understand the origin of the enhanced stability and activity, we investigated the structural evolution of the catalyst during long-term OER operation. During this process, NiFeP undergoes surface reconstruction to form NiOOH/FeOOH, generating highly active sites for oxygen evolution. This in situ transformation facilitates adsorption and desorption of key intermediates (OH*, O*, OOH*), lowering energy barriers and improving reaction kinetics. Previous DFT studies have shown that NiFe oxyhydroxides stabilize these intermediates and optimize surface energetics, which accounts for the enhanced OER performance.^23,69^ The observed increase in both activity and stability in our catalysts directly correlates with this dynamic surface reconstruction, highlighting a clear mechanistic link between structural evolution and catalytic efficiency.

The adsorption energy of oxygen-based intermediates (OH*, O*, OOH*) was used to assess the activity throughout the OER and HER processes. These studies illustrate possible surface restructuring of NiFeP-PCF into partial or fully NiOOH–FeOOH or bimetallic oxyhydroxide entities under the harsh alkaline environment and oxidative nature of the electrolytes.

## 4 Conclusions

In summary, electrospun bimetallic NiFeP-PCF showed great promise as an electrocatalyst candidate for seawater splitting. The block copolymer-derived porous carbon nanofibers were conductive and had a high surface area, facilitating efficient charge transfer and providing active sites that enhanced their electrocatalytic performance. In addition, the bimetallic synergy, along with the component advantages and in situ phosphating process, bolstered durability and corrosion resistance, enabling its effectiveness in saltwater conditions. NiFeP-PCF showed remarkable OER and HER performance in an alkaline electrolyte, requiring minimal overpotentials of 320 and 145 mV, respectively, to achieve a current density of 100 mA cm^–2^. A symmetric electrolyzer using two NiFeP-PCF electrodes required only a cell voltage of 1.8 V and exhibited robust stability over 200 h at 100 mA cm^–2^. The electrolytic cell, utilizing NiFeP-PCF electrodes, effectively powered alkaline seawater electrolysis with exceptional long-term stability, achieving a Faradaic efficiency of 97.8%. The work herein shows immense potential for the economical synthesis of efficient electrocatalysts, paving the way for large-scale hydrogen production.

## Supplementary Material



## Notes

Experimental section, characterization of synthesized block copolymer, SEM images, pore size distribution and electrochemical characterization data (CV, LSV, EIS etc.) of various samples (PDF)
