# Supplementary material for: Seawater Splitting Using NiFeP-Embedded Porous Carbon Fibers
Source: ACS Appl Eng Mater. 2025 Nov 7;3(11):4167–77. doi: 10.1021/acsaenm.5c00785 (PMC12670422; doi:10.1021/acsaenm.5c00785)
Supplement: Supplementary file 1 [file em5c00785_si_001.pdf]

Supporting Information

Seawater Splitting Using NiFeP-Embedded Porous Carbon Fibers

Akshara Paras Parekh[1], Ashish Kumar Yadav[2], Brendan Whitfield[1], Yue Zhang[1], Arshad Aijaz[2], Guoliang Liu[1,3,4*]

[1]Department of Chemistry, Virginia Tech, Blacksburg, VA 24061, USA

[2]Department of Energy & Human Sciences, Rajiv Gandhi Institute of Petroleum Technology, Jais, Amethi, UP-229304, India

[3]Macromolecules Innovation Institute, Virginia Tech, Blacksburg, VA 24061, USA

[4]Division of Nanoscience, Academy of Integrated Science, Virginia Tech, Blacksburg, VA 24061, USA

*Corresponding Author. E-mail address: gliu1@vt.edu

Contents

Part 1: Experimental Section

Part 2: Additional characterization of NiFeP/PCF

Part 3: Electrochemical Studies

Part 4: Characterization after durability test

Part 1: Experimental Section

1. Electrochemical Measurements

1.1 OER and HER

A typical three-electrode test was conducted using an Autolab-PGSTAT302N electrochemical analyzer for all oxygen evolution reaction (OER) and hydrogen evolution reaction (HER) tests, which were performed in 1 M KOH and phosphate-buffered saline (PBS, pH 7) solutions. Ag/AgCl was utilized as the reference electrode, a graphite rod functioned as the counter electrode, and glassy carbon electrodes were employed as the working electrodes for catalyst loading. All electrochemical experiments were performed at room temperature, following the use of cyclic voltammetry (CV) to stabilize the catalyst. Using the equations $E_{RHE} = E_{Ag/AgCl} + 0.098 + 0.0591 \times pH$ for each potential value, the potential corresponding to the reversible hydrogen electrode (RHE) was determined. Here, $E_{Ag/AgCl}$ stands for the measured potential on the electrochemical workstation.

The working electrodes were prepared as follows: first, 5 mg NiFeP/PCF powder was dissolved using ultrasonic treatment in 960 μL of ethanol and 40 μL of 5% Nafion ionomer solution to create the catalyst ink. Subsequently, 40 μL of the resulting catalyst ink was applied onto the glassy carbon electrode's surface, which was dried at room temperature. About 1 mg/cm^2 of catalyst was the equivalent catalyst loading. The electrocatalytic activity of Pt/C (20%) and commercial IrO_2 was compared under the same conditions and with the same catalyst loading. Using an amplitude of 5 mV, electrochemical impedance spectroscopy (EIS) was conducted in the frequency range of 10^{-1} to 10^6 Hz. OER was studied using linear sweep voltammetry (LSV) at a scan rate of 5 mV s^{-1}. The following formula yields the Tafel slope: $\eta = a + b \log j$, where η, a, b, j, represent overpotential, intercept, the Tafel slope, and current density, respectively. Additionally, chronopotentiometric (CP) measurements were carried out at a current density of 100 mA cm^{-2} for 100 h to evaluate the catalyst durability during OER and HER, and for 200 h to evaluate seawater splitting. Cyclic Voltammograms at different scan rates (20 to 120 mV s^{-1}) were acquired in the non-faradaic zone to determine the C_{dl} values. ECSA is calculated according to the equation ECSA = C_{dl} / Cs, where Cs = 0.04 mF cm^{-2}. Additionally, EIS measurements were carried out in the frequency range of 100 kHz to 0.1 Hz at an AC amplitude of 5 mV, with a potential of 0.626 V (versus Ag/AgCl). In a two-electrode setup, the anode and cathode for total water splitting were NiFeP/PCF. The electrode area was set to 1 cm^2 for all analyses and measurements.

2.2 Turnover frequency (TOF) calculation

The turnover frequency (TOF, s^{-1}) for OER was calculated with the following equation:

$$\textbf{TOF} = \frac{|J| .\ A}{4F\ .\ n} \quad \textbf{(S1)}$$

The TOF for HER was calculated with the following equation:

$$\textbf{TOF} = \frac{|J| .\ A}{2F\ .\ n} \quad \textbf{(S2)}$$

where A is the geometric area of the working glassy carbon electrode (0.07 cm^2), F is the Faraday constant (96485 $C \cdot mol^{-1}$), and n is the number of active sites (mol), and |J| ($A.cm^{-2}$) is the current density at a fixed voltage during the LSV measurement. The related electron transfer values are either 2 or a factor of 4. The previously published methods were utilized to estimate the number of active sites (n).[1-3] Using a cyclic voltammogram in 1.0 M PBS solution and a potential range of -0.2 to 0.6 V (vs. RHE) at a scan rate of 50 $mV \cdot s^{-1}$, the catalyst was initially analysed. The acquired CV curve can be used to compute the charge Q, which is proportional to the number of active sites (n).

$$n = \frac{Q}{2F} = \frac{I\ .\ t}{2F} = \frac{I.V/u}{2F} = \frac{S}{2F\ .\ u}$$

where S is the integrated effective area in CV recorded, I is the current (A), V is the voltage (V), and u is the scan rate (50 $mV \cdot s^{-1}$).

2.3 The exchange current density (j_0)

The Tafel slope was calculated according to the Tafel equation as follows: $\eta = b \log |j| + a$, where η is overpotential (V), j is current density ($mA \cdot cm^{-2}$), and b is the Tafel slope ($mV \cdot dec^{-1}$). The exchange current density can be obtained by extrapolating the Tafel curve to zero overpotential (exchange current density $j=j_0$ at $\eta=0$ V).

2.4 The Faradaic efficiency (FE)

The Faradaic efficiency was calculated by comparing the experimentally produced gas volume with the theoretically calculated one:

$$FE = V_{experimental} / V_{theoretical}$$

The experimental volumes of H_2 or O_2 were measured by drainage. The theoretical volume can be calculated using the formula:

$$V_{theoretical} = \frac{I.t.Vm}{n.F}$$

where I is the electrolysis current, t is the electrolysis time, Vm is the molar volume of H_2/O_2 of gas (24.5 L mol^{-1}, 298 K, 101 kPa),12 n is the number of electrons required for one molecule of H_2 or O_2, and F is the Faraday's constant (96485 C/mol).

Part 2: Additional characterization of NiFeP/PCF

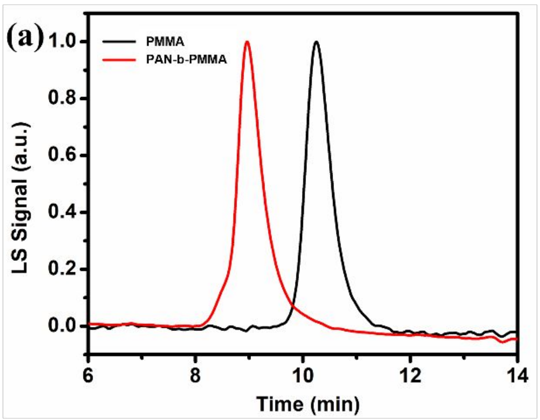

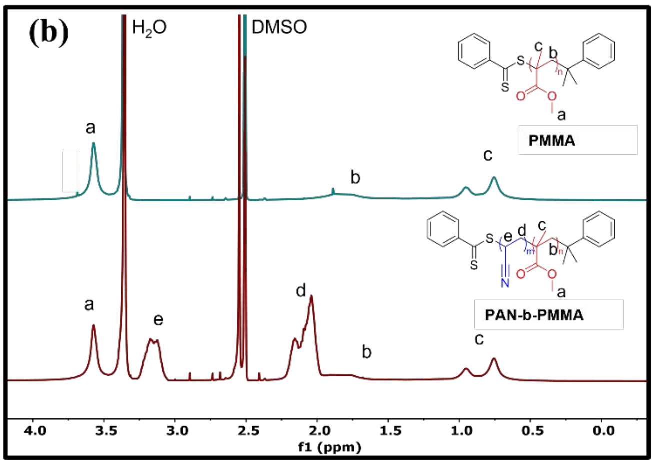

Polymer	Molecular weight (kDa)	PDI
PMMA	46	1.09
PAN-*b*-PMMA	158	1.13

Figure S1. Characterization of the synthesized block copolymer PAN-*b*-PMMA (a) SEC traces and (b) NMR spectra.

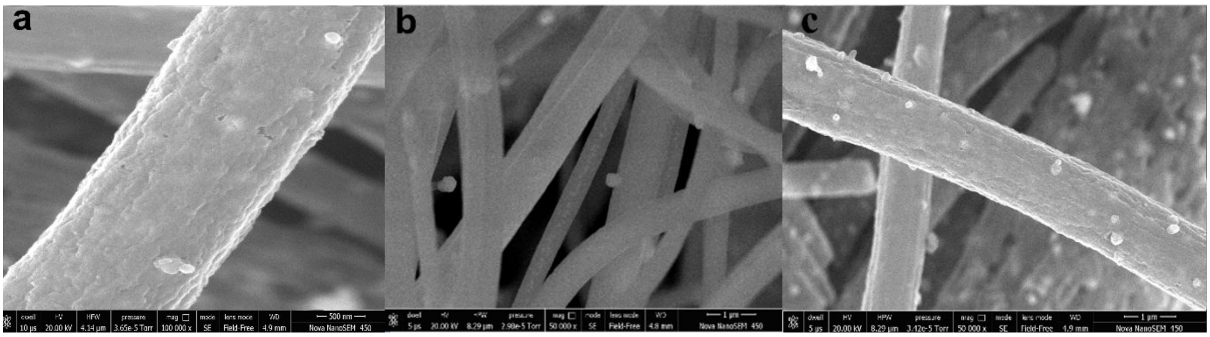

Figure S2. FE-SEM images of a) NiP-PCF, b) FeP-PCF, and c) NiFe-PCF.

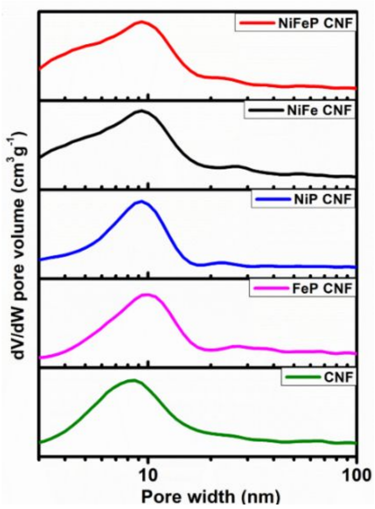

Figure S3. Pore size distribution of PCF, FeP-PCF, NiP-PCF, NiFe-PCF, and NiFeP-PCF catalysts.

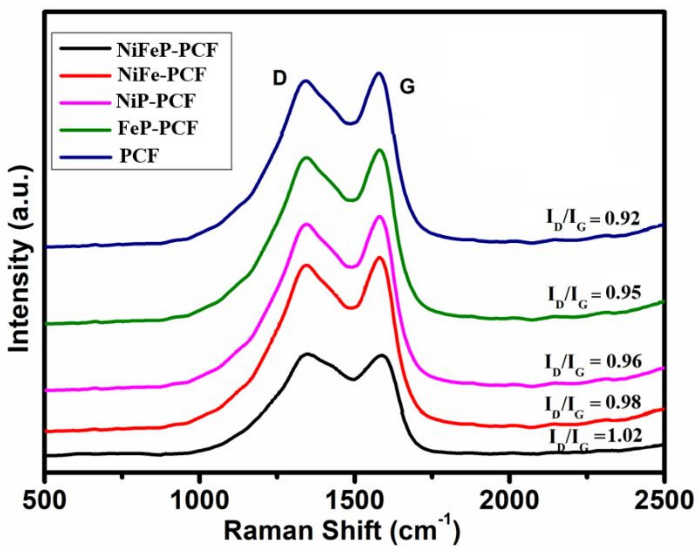

Figure S4. Raman spectra of PCF, NiP-PCF, FeP-PCF, NiFe-PCF, and NiFeP-PCF.

Part 3: Electrochemical Studies:

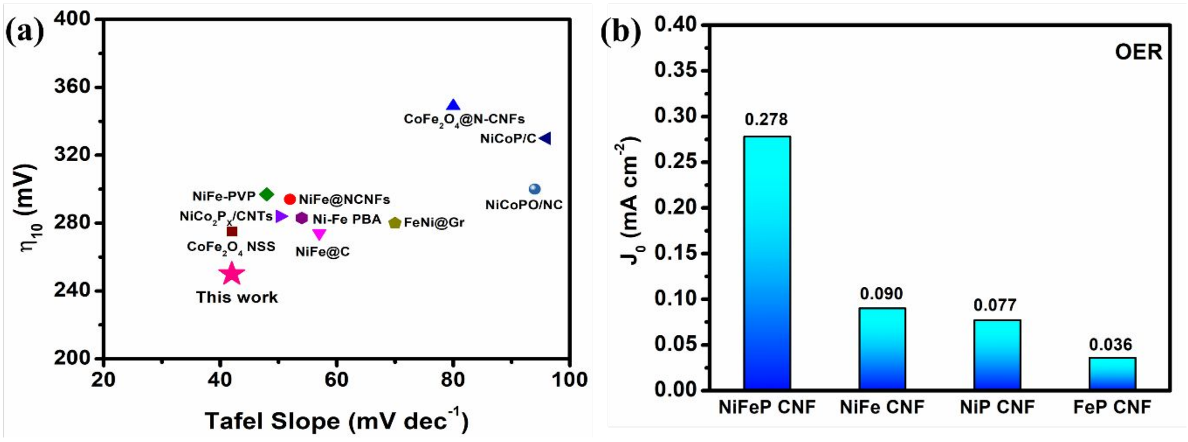

Figure S5. OER in 1.0 M KOH: (a) comparison plot and (b) current exchange density.

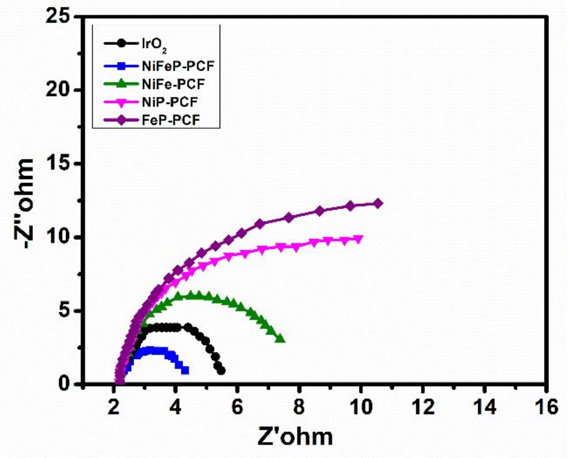

Figure S6. Nyquist Plot for OER in 1.0 M KOH.

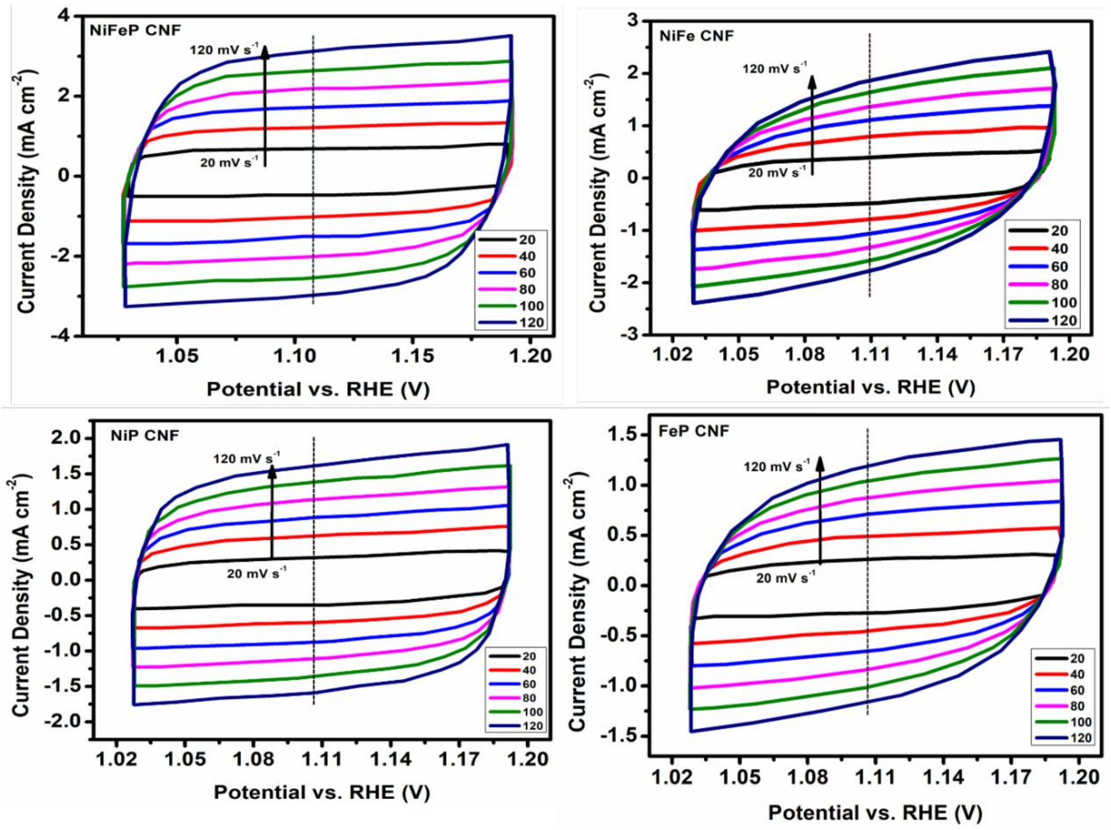

Figure S7. CV curves of NiFeP-PCF, NiFe-PCF, NiP-PCF, and FeP-PCF at different scan rates from 20 to 120 mV s^{-1} for OER in 0.1 M KOH.

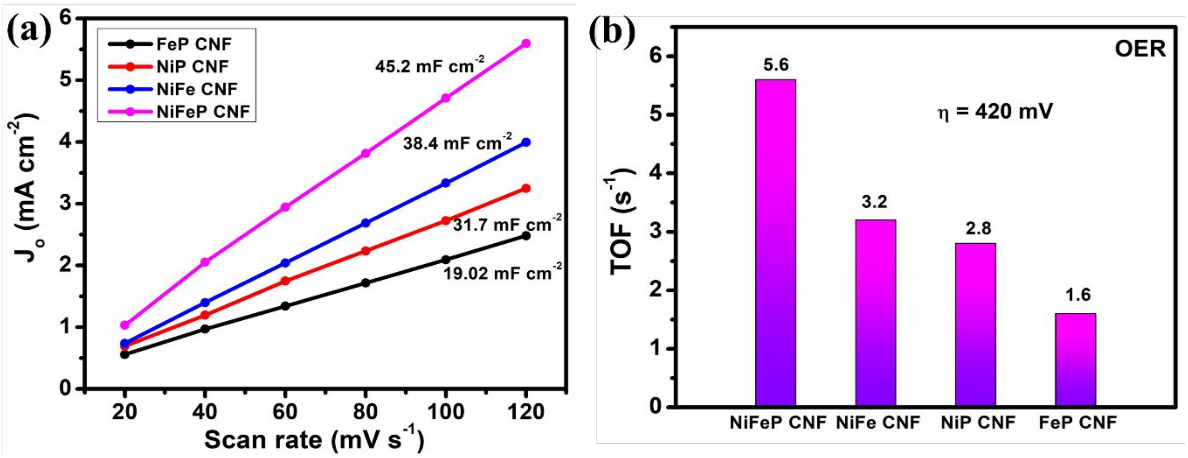

Figure S8. OER in 1.0 M KOH: (a) ECSA and (b) TOF.

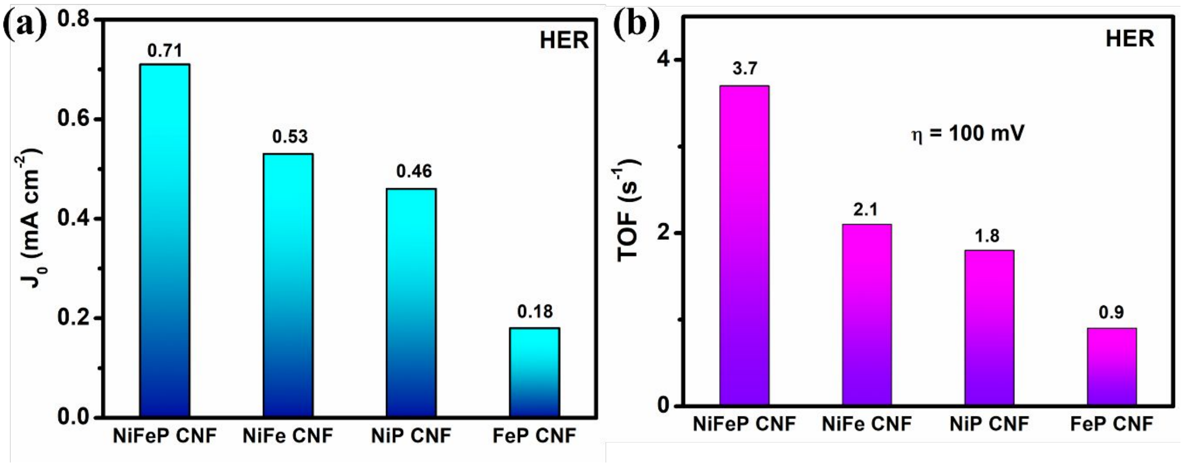

Figure S9. HER in 1.0 M KOH: (a) current exchange density and (b) TOF.

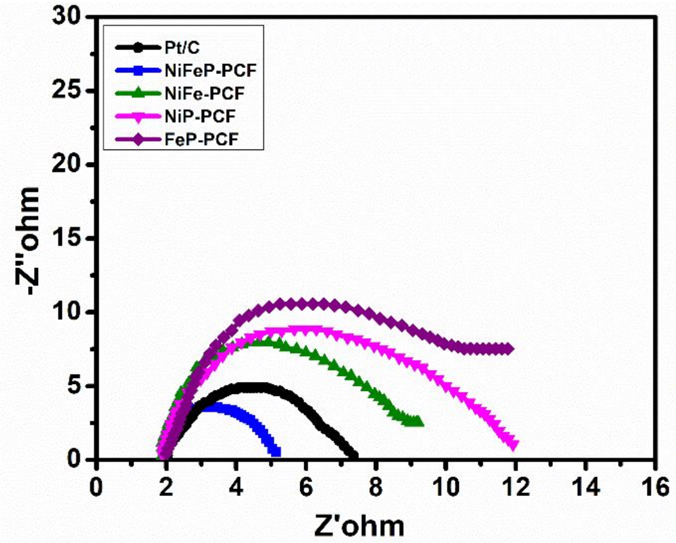

Figure S10. Nyquist Plot for HER in 1.0 M KOH.

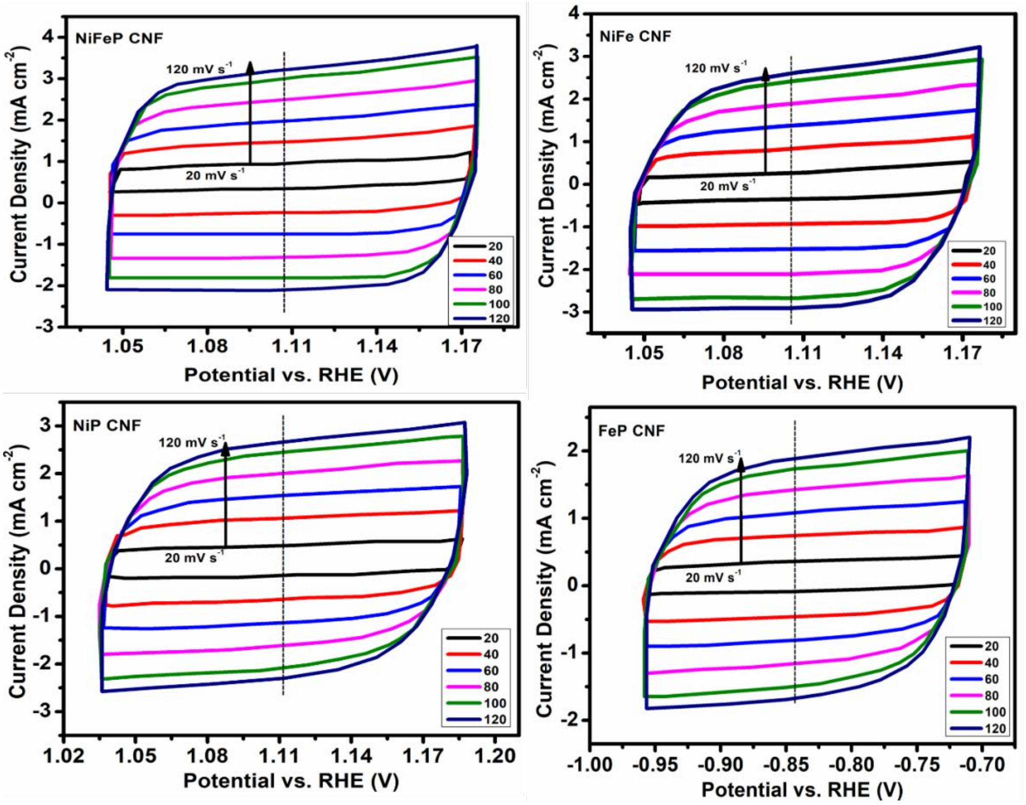

Figure S11. CV curves of NiFeP-PCF, NiFe-PCF, NiP-PCF, and FeP-PCF at different scan rates from 20 to 120 mV▪s^{-1} for HER in 0.1 M KOH.

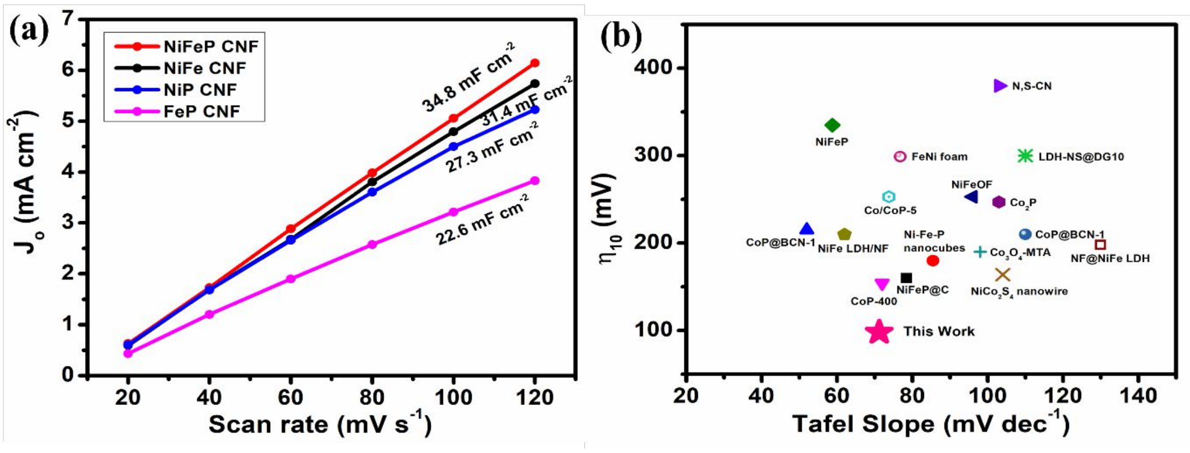

Figure S12. HER in 1.0 M KOH (a) ECSA and (b) comparison plot.

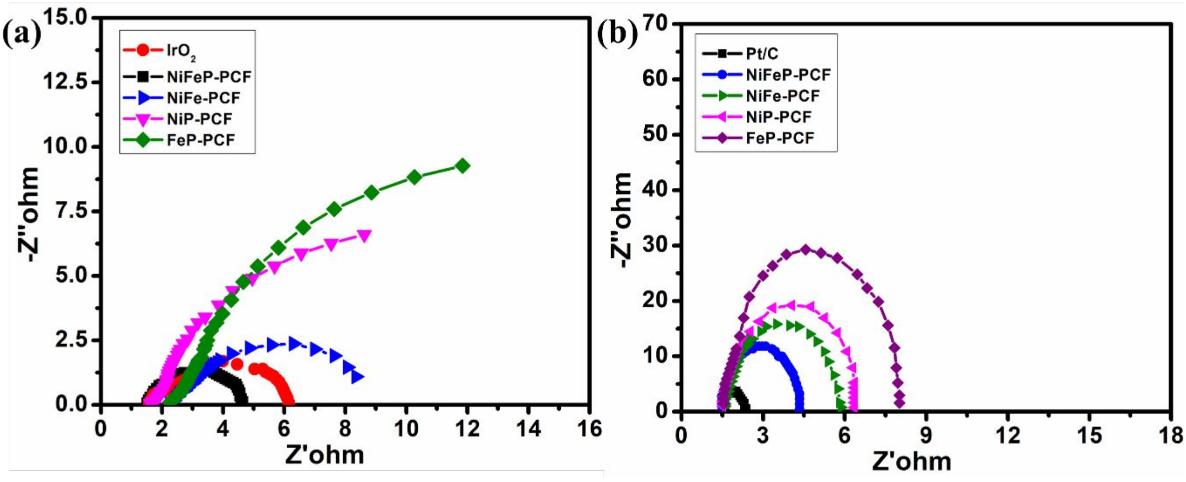

Figure S13. PBS EIS in 1.0M KOH: (a) OER and (b) HER.

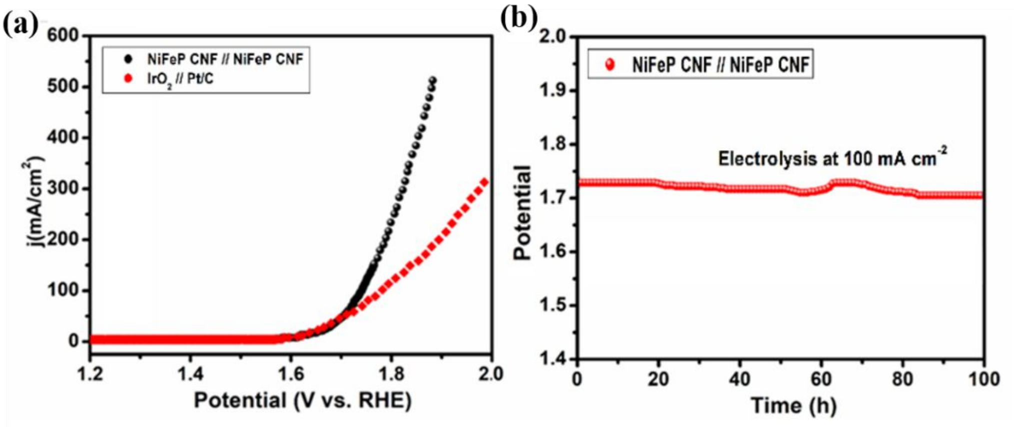

Figure S14. (a) Polarization curves of NiFeP CNF // NiFeP CNF and IrO_2 // Pt/C for overall water splitting in PBS electrolyte. (b) Chronopotentiometric stability test of NiFeP CNF // NiFeP CNF at 100 mA cm^{-2}

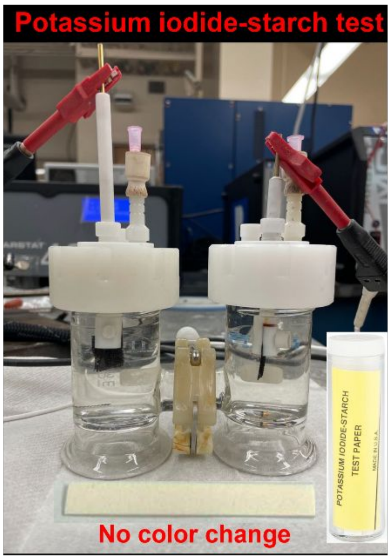

Figure S15. Chlorine precipitation study after 100h of stability testing

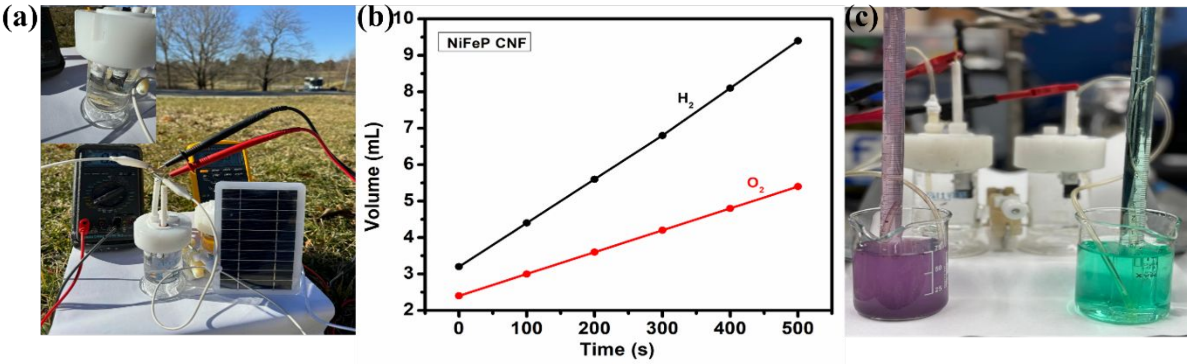

Figure S16. (a) a solar panel-integrated overall water-splitting with the output voltage of 1.566 V, (b) Plot of the displaced volume of water by produced H_2 (purple) and O_2 (green) with time. (c) Photograph of the setup for the measurement of Faradaic efficiency in overall water splitting in seawater.

Part 4: Characterization after durability test

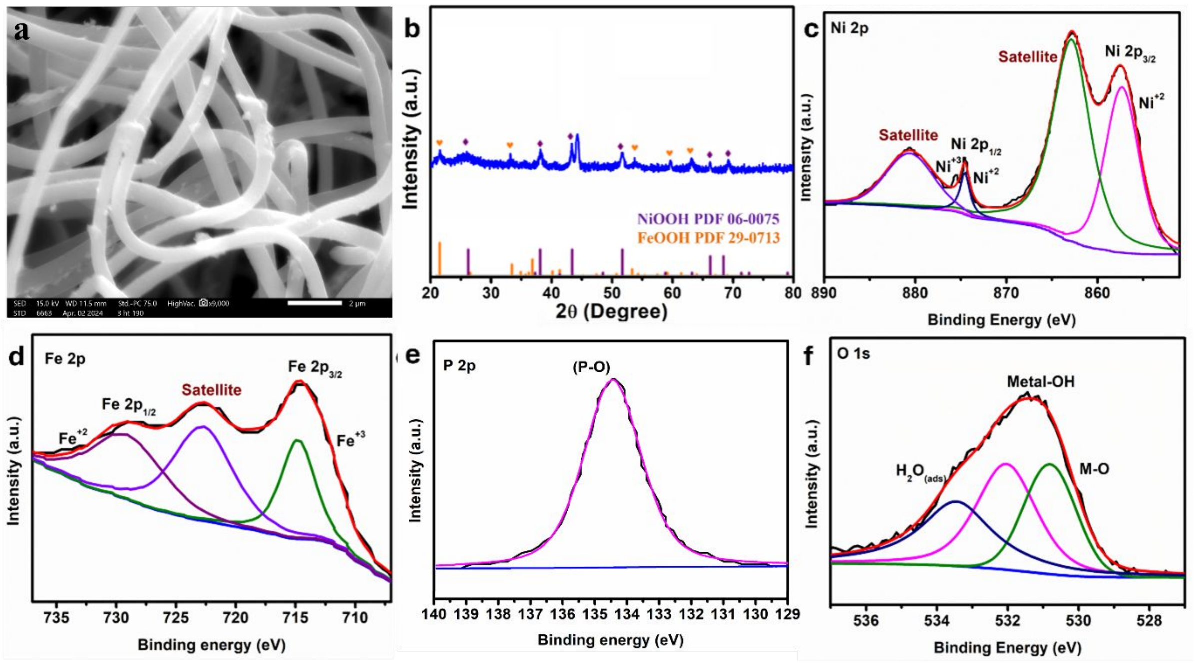

Figure S17. (a) SEM image of NiFeP-PCF after durability test, (b) XRD pattern of the NiFeP-PCF after durability test, (c-f) XPS spectra of NiFeP-PCF after durability test.

References:

(1) Gao, W.; Yan, M.; Cheung, H.-Y.; Xia, Z.; Zhou, X.; Qin, Y.; Wong, C.-Y.; Ho, J. C.; Chang, C.-R.; Qu, Y. Modulating electronic structure of CoP electrocatalysts towards enhanced hydrogen evolution by Ce chemical doping in both acidic and basic media. *Nano Energy* **2017**, *38*, 290-296. DOI: https://doi.org/10.1016/j.nanoen.2017.06.002.

(2) Li, J.; Yan, M.; Zhou, X.; Huang, Z.-Q.; Xia, Z.; Chang, C.-R.; Ma, Y.; Qu, Y. Mechanistic Insights on Ternary Ni2−CoP for Hydrogen Evolution and Their Hybrids with Graphene as Highly Efficient and Robust Catalysts for Overall Water Splitting. *Adv. Funct. Mater.* **2016**, *26* (37), 6785-6796. DOI: https://doi.org/10.1002/adfm.201601420.

(3) Vigil, J. A.; Lambert, T. N.; Christensen, B. T. Cobalt phosphide-based nanoparticles as bifunctional electrocatalysts for alkaline water splitting. *Journal of Materials Chemistry A* **2016**, *4* (20), 7549-7554.
